# Collectanea Medica, Consisting of Anecdotes, Facts, Extracts, Illustrations, Queries, Suggestions, &c.

**Published:** 1815-03

**Authors:** 


### COLLECTANEA MEDICA,

CONSISTING OF

### ANECDOTES, FACTS, EXTRACTS, ILLUSTRATIONS, QUERIES, SUGGESTIONS, &c.

RELATING TO THE

*History or the Art of Medicine, and the Auxiliary Sciences.*

WE make no apology for the length of the following extract. The work in which we found it is not medical, and therefore less likely to fall in the way of many of our readers. The passage transcribed relates a well-ascertained fact, highly interesting to the pathologist, and, as far as our inquiries go, no where else to be met with. We have another reason for inserting it at this time, which we shall mention at the close.

*Gôitres of the Valley of Luchon. History of the Cagots.—From Ramond's Travels in the Pyrenees; translated by Mr. Gold.*

"When the ingenious observer, to whom we owe the essay upon the mineralogy of the Pyrenees, passed through the valley of Luchon, he was struck with the sight of a great number of persons afflicted with considerable gôitres, to whose deformity there was joined an air of stupidity, still further increased by an indistinct articulation. He remarked in these degraded beings a livid and sallow complexion, a weak habit of body, and such apathy, as to give them, says he, an aptitude only for repose. To describe these unfortunate beings, is to describe the Cretins; and the Valais would, in this respect, have no further advantage over the valley of Luchon, than in its being able to produce a greater number of these miserable creatures.

"But such deplorable superiority is not on the side of the Valais. In the southern parts of France, this unfortunate portion of the human race is very widely extended. In the valley of Luchon, their state of beggary brings them more into view; but they are found in the valley of Aure, in that of Bareges, in Bearn, and even in Navarre. These latter exist in the most retired places only, but when seen, exemplify a degradation, a dullness, and stupidity, which even the imbecility of the Cretins of the Valais does not surpass; and which deprives them of the last remains of the intelligence of man, together with the last traces of his figure.

"It might be readily imagined, in observing this sad conformity of condition, that the causes of degradation in both countries should be similar, and that to explain the Cretinism of the Alps, would be to explain the Cretinism of the Pyrenees. But in vain should we endeavour to apply the same systems to the same fact. The Cretins of the Pyrenees occupy the northern vallies of the chain, are found in extensive basins, on an open soil, in a dry and temperate atmosphere, and are in the habit of drinking only fresh and pure waters. Every thing conspires to forbid the inferences of analogy. It is to the south that are found the Cretins of the Valais, of Savoy, of Piedmont; it was to the south, therefore, that I should have found them in the Pyrenees, in those narrow Spanish vallies, where the rays of the sun, reflected in all directions from naked rocks, concentrate a stifling heat; and where, in the vitiated air, are suspended unwholesome fluids, dissolvable only from such an extraordinary degree of expansion; in those southern vallies also, where, as in the Alps, the declivities are more abrupt, the rocks more steep, and the mountains more decrepid, should I have found those waters which wash a surface of imperfect slate and schists, the calcareous particles of which are dissolved by means of a sulphuric or carbonic acid, and deposited in the vessels of those who are obliged to drink them. This latter cause of Cretinism, indeed, may probably exist in some of the northern vallies, but it cannot operate as a general cause; for Bercugnas, which is watered by the Go, possesses persons afflicted with gôitres, while Bagneres, which it waters also, has not any, and Saint Mammet, which it does not water, has even more than Bercugnas.

"Another argument remained in favour of the system which makes the stupidity of the inhabitants of the Pyrenees accord with that of the elevation of their vallies, and their distance from the sea. But this explanation, however plausible at its first appearance, and whatever probability it assumes from the consideration of the agility of the Basque, when opposed to the dullness of the inhabitants of the valley of Luchon, loses all its force when the inhabitants of the southern and eastern parts of the Pyrenees are examined, and does not at all account for the Cretins of Bearn and Navarre. Habituated, on the other hand, from experience and former observations, to look upon force and agility as the lot of the inhabitants of elevated mountains, and having uniformly observed that sloth, infirmity, and Cretinism, by no means affect a lofty situation, I was forced, not only to allow that Cretinism is an accident, independant of such circumstances, but to consider the different degrees of vivacity, of force, and of agility, possessed by the people of the

Pyrennees,

Pyrennees, as the appendage of particular races of men, rather than as the production of the soil or climate.

"My own observations, therefore, could throw no light upon the subject; and the best-informed persons whom I consulted, could not resolve the problem in a more satisfactory manner. I was obliged, then, to add another fact to the numerous list of those which show that similarity of effect does not always depend upon identity of cause, when, from my commerce with the people of the country, I found the nature of the question changed entirely, and discovered that the Cretins of the valley of Luchon were of the unfortunate race of the Cagots.

"But this information I drew from the inhabitants as a kind of confession, and could scarcely overcome the shame with which it was made known to me. They informed me that their vallies contained a certain number of families, which, from time immemorial, had been regarded as making part of an infamous and accursed race; that the individuals composing such families had never been reckoned among the number of their citizens; that they were every where disarmed; that they were permitted no other occupation than that of wood-cutters, or carpenters, an occupation become ignoble, like themselves; that they derive one of their common appellations from this occupation; that such appellation is infamous, because they bear it; that, as carpenters, they are every where obliged to march the first in case of fire; that, as slaves, they are forced to perform in every community such services as are reputed ignominious; that misery and disease are their uniform portion; that they are commonly afflicted with gôitres; that their miserable habitations are removed into retired spots; and that, if at present the inhabitants of the country have less aversion to them, and if milder manners have a little softened the rigour of their old condition, there is still between the two races nothing in common, no commerce, and no alliance which is not, in the villages that witness it, regarded as an object of scandal.

"I thus discovered myself to be in the midst of this people of slaves, whose origin is lost in the stormy night of the first ages of our monarchy. I beheld this rejected cast, upon which so much has been written, without dispersing the darkness which covers the motives of so strange a proscription, and the individuals of which it was in vain to question, as, together with the rights and dignity of man, they have lost their traditions, and exist at present only as a monument of the miseries of an age which has transmitted nothing to us but what is odious or deplorable.

"What fact, indeed, is worthier of exciting the curiosity of the historian, and the pity of the philosopher, than the existence of this unfortunate people, whose miserable descendants are scattered along the ocean, from the north to the very south of France, and have every where been the objects of the same aversion, the victims of the same inhumanity. In the solitudes of Britanny, we find them treated with barbarity from the remotest of times. Scarcely were they permitted, in a more civilized age, to exercise the trades of of rope-makers and coopers. The parliament of Rennes was even obliged to interfere for the purpose of granting them the right of sepulture. At that time we find them known by the name of Cacous, and of Caqueux. The dukes of Brittany had ordained, that they should not appear without a distinctive mark. Towards Aunis, they appear again in the island of Maillezais. La Rochelle is peopled by these Coliberts or slaves. They re-appear under the name of Cahets in Guiyenne; and, in Gascogny, are banished into the morasses and heaths of the country. In the two Navarres, they are sometimes called Caffos: they are named so in the ancient For, compiled about 1074. Lastly, they are discovered in the mountains of Bearn and Bigorre, in the four vallies, and the county of Cominges. There they are the Cagots or Capots, who, in the eleventh century, were given away by will, were sold as slaves, and reputed to be infected with leprosy and contagion, so much so, as to be obliged to enter the church by their separate door, and use their particular font, and seats, which were placed apart. In many places it appears that the priests would not admit them to confession; and, as we see by the ancient For of Bearn, an act of favour was thought to be granted them, when seven of them were admitted as an equivalent to the testimony of one free citizen. Even as late as the year 1460, the states of Bearn required that they should be forbidden to walk in the streets barefooted for fear of infection, and that they should wear upon their clothes their ancient distinctive mark, the foot of a goose or duck.

"The learned, the common people, and these wretches themselves, are equally ignorant of the sources of such hatred, and the time which gave it birth. The conjectures of the one, and the fables of the other, have this in common, that they go back alike to the darkest epochs of our history, and advert alike to the ravages of the leprosy; but, before the attempt of M. de Gebelin to account for the astonishing conformity of fortune and name which embraces people divided from each other by such distances, there was no approximation of them even thought of; and the possibility of such approximation must henceforth be the touchstone of every system which has for its object to explain the origin and fate of these hordes, if so they may be called.

"In fact, the Cagots of all France have a common origin. The same event has confined them all in the most remote and desert spots; and, whatever this event may be, it must be such as will account for every thing; it must be great and general; must have impressed at once upon the whole of France the same sentiments of hatred, have marked its victims with the seal of the same reprobation, and have disgraced the race, and all its subdivisions, with the opprobrium of a name which every where awakened the same ideas of horror and contempt.

"But little reliance, then, can be placed on the account which makes them to have been descended from lepers, banished the society of men. Lepers have been banished or confined, but have neither been sold, nor left by will, nor given away. And even

 should should it be true, that the Cacous of Britanny were the white lepers of the time of Ambrose Paré, he may have described their state without having proved any thing respecting their origin.

"Neither does it appear more probable that they are the descendants of the Gauls, reduced to such a state of degradation by the barbarians who usurped the place of the Romans. We are perfectly well assured, that, under the Goths and the Franks, the condition of the Gaul and Roman had no relation whatever with a state of slavery and infamy, and what we have to explain is aversion, not tyranny: the slave is oppressed, but the Cagot was driven away: we must account for motives of contempt and vengeance, not the despotism of a conqueror. Such victory as may have terminated the conflict of two nations, equally ferocious, and inflamed against each other by a long state of rivalry; the invasion of one barbarian punished by another barbarian; the re-action of the oppressed against the oppressor, at last completely disarmed; bloody combats, disastrous defeats; such only could have been the sources of the hatred and fury which could have given rise to miseries like those which we behold.

"But where are we to choose, or on what can we fix in such a period? What combat was the most bloody? What nation the most unfortunate? In what way can we distinguish between the traces of the conqueror and those of the conquered, in such a series of confusion?"

The writer then offers a number of opinions concerning the race of natives or barbarian invaders who, by conquest or some other cause, became thus the degraded slaves or outcasts of the whole country between the Alps and Pyrenees, and are found the most numerous in the vallies formed by those mountains. Here we shall not follow him, because all ends in conjecture; and the only conclusion, if any, is, that they were a mixed race of subdued Goths, of Alans, and of Arians: after which he assigns the following as the probable causes of their degeneracy.

"The refusal of the sacraments of the church, and of the right of burial, was the natural consequenee of the resentment of the clergy, who had been for a long time persecuted. Being Arians, they were banished from the communities of men, because they were heretics, and not because they were lepers. They became lepers when a successive degeneration, the natural fate of a race devoted to poverty, had naturalized disease among them. By degrees, indeed, they fell in with the faith of the church; but the faith of the church could not regenerate their race in its pristine purity. They ceased to be Arians without ceasing to be lepers, and ceased to be lepers without ceasing to be the prey of those diseases which are engendered from a vitiation of the blood.

"The feudal institutions, which became the government of the barbarians, as they sunk still deeper into barbarism, were no longer contented to divide the land with the cultivator, but appropriated the person together with the soil; and the Cagot, among a race of slaves, slaves, because a slave of the very lowest condition. The common people, indeed, re-acquired, in some degree, their rights; but the Cagot for his part had only the shadow of liberty, and remained in a state of dependence, so much the more miserable, as in the number of his tyrants he no longer had a master who provided for his wants.

"Such, then, is the destiny of a nation which overturned and founded empires; upon whom the last remains of its particular opinions have drawn down a greater vengeance than even the remembrance of its usurpations. The whole nation of the Goths, exterminated in battle, or mingled with the inhabitants of the country, have disappeared from France and Spain; a proscribed cast is all that we can find of them; and under features, degraded by twelve centuries of misery, is it that the last remains of Gothic pride are buried. A livid complexion, deformity of feature, the stigmata of disease; such are now the characteristics which distinguish the posterity of a race of conquerors; for every vestige of their former appearance must now be extinct, excepting, perhaps, some few traces of a foreign make, which the degradation of their species has not entirely destroyed, because such configuration can only yield to the mixture of a people with others, and not to their misfortunes.

"I have seen some families of these unfortunate creatures: they are insensibly approaching the villages from whence they have been banished. The side doors by which they enter the churches have become useless, and some degree of pity is mingled at length with the contempt and the aversion which they formerly inspired; notwithstanding this, I have met with retreats in which they still apprehend the insults of prejudice, and await the visits of the compassionate. I have found among them the poorest beings, perhaps, which exist upon the face of the earth. I have seen among them creatures whom society has not been able to render so vile as it has attempted to make them. I have met with brothers who loved each other with that tenderness which is the most pressing want of isolated men. I have seen among them women, whose affection had a somewhat in it of that submission and devotion which are inspired by feebleness and misfortune. And never, in this half annihilation of these beings of my species, could I recognise, without shuddering, the extent of the power which we may exercise over the existence of our fellow; the narrow circle of knowledge and of enjoyment within which we may confine him; the smallness of the sphere to which we may reduce his perfectibility. Alas! if there exists the benevolent being, who, in comparing man with his actions, and beholding before what objects of fear and desire he is prostrated, who, in imagining what he might be, and observing what he is, the sport of his legislators and conquerors, has not started as from the dreams of a fearful sleep, and sprung towards a futurity unapproachable by violence or error, towards a futurity where he who created us to feel has reserved for the unfortunate, and the insensate, that inviolable portion of happiness which they should should have enjoyed,—of truth, which they should have known; if so many miseries for such a mind have not their recompence, and so many tombs are shut without return on so many who have been unfortunate, alas, how much is such a being to be pitied!"*

Such is the description of a race of men dwelling among the most refined of nations, and in a country more visited, and the inhabitants of which have been oftener described, than of any other part of the globe. Yet, though their existence as a separate race is traced with certainty for at least 1500 years, till now they have never been known by name, nor their origin even conjectured. Of the gôitre of Switzerland, indeed, every traveller gives us some account; but we do not recollect more than one writer who has conjectured that this deformity has become so general in those regions by a seclusion from the rest of mankind, and by perpetual intermarriages. We are not certain that this writer ever saw the people concerning whom he offers this conjecture, and can hardly suppose he would have taken his opinions from M. Ramond's work without acknowledging it; yet it is fair to remark, especially as our pages are open to explanation, that the *translation* from which we have transcribed is dated 1813. At what time the original was published, we are entirely ignorant.

---

### *Account of a Fall of Uranolytes (Aërolites) near Agen; by* M. De Saint Amans.

On the 5th of September 1814, a few minutes before mid-day, the wind being northerly, and the sky perfectly serene, a violent detonation was heard in the communes of Montpezat, Temple, Castelmoron, and Montelar, situated in the first, second, and fourth *arrondissemens* of the department of the Lotand Garonne. This unusual detonation was immediately followed by three or four others at an interval of half a second successively; and finally, by a rolling noise at first resembling a discharge of musketry, afterwards the rolling of carriages, and finally, that of a large building falling down. These detonations, which took place towards the centre of the department, were heard with more or less intensity within a circle of several leagues. Thus at Agen, four leagues off, they were sufficiently strong to alarm some persons, and the concussion of the air was such as to shake the doors and windows of certain houses; while at Paymirol, two leagues to the eastward of Agen, these effects were less sensible; and at Mezin, St. Macaire, Basas, and Condon, situated five or six kilometres from the focus of the explosion, it was heard in a very indistinct manner.

At the end of this phenomenon, which, considering the state of the atmosphere, could not be occasioned by any storm, we were led to expect a fall of these meteoric stones, which has always been preceded by similar detonations. We soon learned, in fact, that this fall, accompanied by a kind of lightning, had taken place in the communes above named. From the written and verbal reports ports which have reached us, the number and volume of these stones appears to have been considerable. Some were sent to the prefect, who has communicated them to the minister of the interior: others were distributed among the curious in various parts of France, while many were picked up by the peasants and venerated as reliques. Two are mentioned as weighing eighteen pounds each. It seems that they were not found warm at the moment of their fall: the heaviest were sunk into a compact soil to the depth of eight or nine inches, and one of them rebounded three or four feet from the ground. It is added, that these stones fell obliquely, making an angle of from 65 to 70 degrees with the horizontal line; finally, that they diverged in their fall, affecting various directions in the different communes where they fell. Like all those which have come from similar meteors, they appeared to be fragments of more considerable masses, and are perfectly homogeneous. All the specimens of these stones which I saw, present no character to the eye which distinguished them from those which I have hitherto had occasion to examine, or which I have in my cabinet: they merely seemed to be more friable and more porous than the latter. I have remarked in some fragments globulous bodies, similar to those which Mr. Howard found in a great quantity in the uranolites of Benares, and which are composed, according to him, of abundance of silex with a little oxide of iron. We observed also in the interior of those stones, that the pyrites which they contain are sometimes crystallized in a group. All of them are covered externally with a black crust, of the thickness of a quarter of a line nearly, which announces the action of fire, as we see in all the stones of the same kind. Two of our correspondents inform us that one of them exhibits singular impressions at the surface, but it is necessary to verify this.

In fact, of all the peculiarities which the phenomenon presented, the most remarkable is the very simultaneous appearance of a small cloud, which seems to have accompanied the meteor, and even to have preceded it a few seconds. This small white cloud, grayish in the centre, appeared to move with the greatest rapidity over the district where the meteor fell. In other parts, and particularly from the spot where I observed it, it seemed stationary before the explosion. It has been generally admitted that this small cloud had a roundish form. Scarcely was it perceived in the communes where the uranolites fell, when the explosion, accompanied by lightning, was heard. At the very instant the cloud appeared to be divided into three or four parts, which were rapidly precipitated towards the ground, leaving behind them irridations of a blueish colour, and the extremity of which was red. From the position which I occupied, it was seen directly in the north, inclining a little to the north-west. It seemed then to be immoveable; but, at the moment of the detonation, it seemed to advance very rapidly towards the south, forming two points which were prolonged in the sky, and which the peasants unanimously compared pared to long cords. After this sudden movement, the small clouds, which had attained nearly my zenith, considerably diminished, stopped, became immoveable, and ended by being insensibly dissolved at the same place. It cannot be doubted, I think, that the instantaneous appearance of this cloud, insulated in a sky absolutely deprived of all vapour, is connected with the meteor. It has been observed under the same forms nearly in every place where the detonation was heard, and its immoveability, notwithstanding the strong wind which then blew, proves that it must have been very high in the air. We cannot, I think, refrain from regarding it as the produce of the gases emanated from the stony mass, which, when heated by the friction which it underwent in traversing the atmosphere, allowed them to escape under the form of a condensed vapour. The nebulous appearance which resulted must have given rise to several optical illusions on the part of the spectators, who, before the explosion, had no interest in observing it. To those who were close to the place where it fell, it seemed to move with a great rapidity: to those who were, like myself, four or five leagues towards the south, it appeared stationary. In advancing directly opposite to the latter, it must in fact have appeared to them without motion, until the explosion made it assume another form, and until, as it approached their zenith, they must have perceived its progressive motion. This cloud must, therefore, have been the result of the gases developed in the bosom of the mass, which must have in the first place formed around a spherule of vapours, and which, being more and more rarefied, as the mass approached the surface of the earth, must have caused its explosion.

To conclude: this explosion must have been effected, as I have already said, in a high region of the atmosphere, since the wind had not reached the small cloud, and since the fragments of the mass were dispersed, diverging over four communes in a radius of five great quarters of a league. If similar clouds have not always been remarked simultaneously with meteors of this kind, since they have been observed with care, this has arisen from few of those meteors having been seen in such a serene sky, and other clouds must have been confounded with the peculiar cloud which accompanies them.

Here let me direct the attention of my reader for a moment to the term *aërolite*, which is commonly given to meteoric stones. This denomination does not seem to be the best which may be employed. In fact, it is far from certain that these stones are formed in the air or with air. The elevation of the meteor which produces them having been observed to be at least thirty leagues from the surface of the ground, proves that they have nothing in common with the fluid which supports life on the surface of our globe. The name of *uranolyte* has long appeared to me to be better suited to bodies whose origin is unknown to us, but which tend towards the earth through that boundless space in which the stars move, and which is unanimously called the heavens. The term, therefore, which is formed of the Greek words ουρανος and λιθος, deserves the preference to *aërolite*, as being more definite. Some writers have even adopted the term *uranolite*, since the publication of a Memoir which I read to the Society of Agriculture, Arts, and Sciences of Agen, in 1808.—*Annales de Chimie*, tome xcii. p. 25. Oct. 1814.

---

### *Answer to Mr. R. Phillips's Animadversions on Mr. Hume.*

To Dr. Thomson.—(From the *Annals of Philosophy*.)

SIR,

YOUR correspondent, Mr. R. Phillips, affords another example of human imperfection, that we are all "prone to complain" and often to publish too, without any reason or foundation.

Much of his valuable time, and more than nine-tenths of his letter to you, might have been spared, had he only looked into one of the volumes of your own System of Chemistry. There he would have found, that, instead of dashing into a stream of words, studied expressions, and polished sentences, you have, in a single line, said all that was necessary to correct where there was assuredly on my side no intentional plagiarism; for, speaking of the super-sulphate of barytes, you say in a note, "Mr. Hume has also mentioned it, but the fact was well known to chemists." To your decision I have long ago assented, and have also abandoned all claim to priority respecting the super-sulphate of strontian, after I found that Mr. Clayfield, of Bristol, had anticipated my observations.

Mr. R. Phillips is equally unfortunate in the two experiments quoted from M. Sage; he has drawn inferences diametrically opposite to those of all the chemists who have written upon the subject, especially those of France. In these experiments there is neither *water of solution* nor *water of crystallization;* there is no guide to direct future operators to avoid such errors as I shall presently quote; nor is there any room for asserting, that putting a quantity of *carbonate* of barytes into either of the two acids, although it be granted that in such a case it must be converted into a nitrate or muriate, is precisely the same as adding these salts or more particularly their solutions to the respective acids. There is nothing here to invalidate what I have said on the subject; nothing detailed by M. Sage to show the direct meaning and tendency of the instructions which I took the liberty to offer, and which may be thus epitomized:—*That such is the avidity of nitrous and muriatic acid for water, that they will attract even the whole of the water of solution from their respective barytic salts.*

When I published my paper upon this subject, it had been a common practice to ascertain the purity of nitrous acid, and even to purify it, by dropping into it a solution of nitrate of barytes; and this method was pursued by some very eminent professional men, men. I recollect one case in which a gentleman, well known as a chemist and mineralogist, condemned some nitrous acid which had been sent to Sir John St. Aubyn, which, on my proving the error, was not returned. This happened above 14 years ago, and consequently was subsequent to M. Sage's experiments. Indeed, from the letter to you, I have no doubt but Mr. R. Phillips himself has often committed the same blunder.

That M. Bouillon-Lagrange did not benefit by M. Sage's example as he might have done by mine, will be evident to Mr. R. Phillips by the following sentence from Manuel d'un Cours de Chimie: "Ce sel (nitrate de baryte) est utile pour reconnaitre la presence de l'acide sulfurique. On peut s'en servir pour separer cet acide, qui se trouve quelquefois dans l'acide nitrique, et qui empêche de l'employer dans des experiences exactes."

I shall select another case, out of a great many I could name: it is that of Dr. Swediaur, who, as well as M. Bouillon-Lagrange, must have been even personally acquainted with M. Sage, and could not be ignorant of the story of carbonate of barytes in the acids, which may now be called Mr. R. Phillips's fable. In order to divest nitrous acid of sulphuric acid, the doctor prescribes the following plan. Let a solution of nitrate of barytes be added until there be no more precipitation, "donec nihil amplius præcipitetur." I trust this experiment will be closely examined by Mr. R. Phillips, and that he will favour your readers with the result, the *nihil amplius*; and, for my own part, it is of no moment in this experiment whether the acid contain sulphuric acid or not, for I will venture to predict that the experiment will prove equally amusing to Mr. R. Phillips or to any other operator.

Discoveries and improvements in science invariably precede the dates of their being presented to the public, depending on the disposition, convenience, and pursuits, of their authors, and various other causes. In my own case these intervals have generally been of many years, and that particularly of employing *silver* as a test for *arsenic*, and asserting its superior efficacy, was not published until above 20 years after I had discovered it. It occurred to me while examining certain materials used in the preparation of carmine, and its utility was more distinctly evinced in subsequent trials, especially while analyzing a metallic ore belonging to the late Judge Buller. I might indeed appeal to many living witnesses that I am not so forward to tease the public with my writings as Mr. R. Phillips would insinuate. The Journals of the Royal Institution will bear testimony that I can prefer a *private* communication. I am unwilling to bring names forward on this occasion without permission of the parties; but I can recollect one case in point, and not unlike one of the experiments of M. Sage, which took place more than ten years before my remarks on barytes were published. Few philosophers have contributed more effectually to the Philosophical Transactions than the gentleman to whom I now allude, and he probably has not forgotten the short conver-

 sation sation that passed in his library, at that time in the same street and very near to my residence, on our inspecting a vessel containing carbonate of barytes in powder mixed with nitrous acid, and in a state of apparent quiescence. I am persuaded that this gentleman did not then consider me at all ignorant of any thing respecting M. Sage's experiment, or that I did not know in 1792 what I published in 1802 respecting barytes.

The two physicians to whom Mr. R. Phillips alludes, cannot be more respected by him than by me; their reputations stand very high, and most deservedly so; they need no panegyric from either of us, and can fight their own battle; therefore any allusion to them on the present occasion is both irrelevant and intrusive. Such an interposition is more benefitting an hireling than one in pursuit of the truth, and there are various ways of engaging such characters; for even flattery, ambition, malevolence, or jealousy, is often as effectual a stimulus as any thing of a pecuniary nature.

I shall now expect to be told that I have also been forestalled respecting my test for arsenic; that the arseniate of silver, the *brick-red* coloured compound, had been prepared by others; that it had been formed before I was born, having been found in the laboratory of that busy old being Dame Nature; that Henckel, Bergman, and others, can bear testimony to the fact; and that M. Klaproth had frequently got hold of it, analyzed it, regenerated the same compound by means of nitrate of silver; but was so cruel and unlucky as to disregard the silver as a *test*, always preferring the *acetate of lead*, even to the end of the second volume of his valuable Analytical Researches; thus depriving Mr. R. Phillips of another theme for his peevish effusions.

Upon the whole, sir, and from the last paragraph of the letter, I think your correspondent writes more from principles of enmity and revenge than from a desire to improve science; and, as he seems to hold out a threat, I must be prepared to repel such attacks as I may now expect from one who is capable of treating me with so much malice and so little candour.

I remain, Sir, your obedient servant,

Jos. Hume.

*Long Acre; Dec.* 12, 1814.

CRITICAL

